# Wearable photobiomodulation halts thyroid cancer growth by leveraging thyroid photosensitivity

**DOI:** 10.1002/btm2.10734

**Published:** 2024-12-20

**Authors:** Changrui Zhao, Kun Fu, Jiameng Tian, Tian Long, Jianzhong Song, Siyu Chen, Chang Liu

**Affiliations:** ^1^ Department of Endocrinology Nanjing Drum Tower Hospital, School of Life Science and Technology, China Pharmaceutical University Nanjing China; ^2^ Mudi Meng Honors College China Pharmaceutical University Nanjing China; ^3^ Department of Pharmacy The Afffliated Tumor Hospital of Xinjiang Medical University Urumqi China; ^4^ State Key Laboratory of Natural Medicines China Pharmaceutical University Nanjing China; ^5^ Jiangsu Provincial University Key Laboratory of Drug Discovery for Metabolic Inflammatory Diseases (China Pharmaceutical University) Nanjing China

**Keywords:** cell cycle, papillary thyroid carcinoma, photobiomodulation, wearable device

## Abstract

With papillary thyroid carcinoma (PTC) rates rising significantly, concerns about conventional treatments like thyroidectomy and radiotherapy highlight the need for non‐invasive options. Our study explores photobiomodulation therapy (PBMT), which uses specific light wavelengths to evoke cellular responses in PTC treatment. Our research utilized a custom‐designed optical system to investigate PBMT, finding that blue light at a wavelength of 465 nm can safely and effectively inhibit the proliferation of the TPC‐1 PTC cell line by inducing cell cycle arrest. Additionally, we developed a wirelessly powered wearable PBMT device, which is equipped with an advanced light delivery system that ensures precise and consistent dosage. This device designed for optimal patient comfort, effectively suppressed tumor growth in mouse models without adverse effects. PBMT indicates thyroid tissue's light responsiveness as a non‐visual organ. Our study's innovative approach integrates the disciplines of oncology, biophysics, and medical device technology, thereby advancing the treatment paradigms for PTC. This interdisciplinary bridge not only highlights our groundbreaking findings but also paves the way for future research in cancer therapy and photomedicine.

**Translational Impact Statements**

Our study demonstrates that PBMT effectively inhibits proliferation in papillary thyroid carcinoma (PTC), suggesting its potential as a non‐invasive cancer treatment. This approach offers promising clinical applications by leveraging light therapy to target cancer cells while preserving healthy tissue, thereby improving patient outcomes and quality of life.

## 1 INTRODUCTION

Thyroid cancer, specifically papillary thyroid carcinoma (PTC),^1^ is the most common endocrine malignancy,^2^, ^3^ and is often misconceived as benign due to its high survival rate.^4^, ^5^ However, it presents complex challenges, including potential rapid growth, recurrence,^6^ and metastasis,^7^ which may lead to increased cancer‐related mortality.^8^ Moreover, the increased detection of microcarcinomas^9^ has raised concerns about overtreatment and diagnostic uncertainty.^10^, ^11^

Current treatments for PTC are effective but often require the removal of the thyroid, the largest endocrine gland in the body.^12^ These primarily surgical methods can lead to nerve damage,^13^ scarring,^14^ and necessitate lifelong hormone therapy.^15^ Moreover, additional radioactive iodine therapy^16^ increases the treatment burden. Given these challenges and notable gender disparities,^17^ there is a critical need for non‐invasive, patient‐centric therapies, prompting us to explore photobiomodulation therapy (PBMT).^18^, ^19^ PBMT utilizes specific light wavelengths to induce therapeutic responses in cells, with promising minimal systemic side effects.^20^, ^21^, ^22^, ^23^ It emerges as an innovative alternative in the quest for gentler, effective cancer therapies,^24^, ^25^ modulating mitochondrial activity,^26^ enhancing cellular repair,^27^ and reducing inflammation.^28^ Recent evidence of PBMT's anti‐proliferative effects^29^ underscores its potential. Our study delves into PBMT in PTC, aiming for effective, side effect‐free treatments^30^ and improved patient quality of life.^31^, ^32^

PBMT's emerging role in cancer care, particularly for PTC, is still in its infancy. Although previous studies have demonstrated PBMT's therapeutic potential, its effectiveness in thyroid carcinoma lacks direct evidence. Our study seeks to fill this gap by investigating PBMT's effects and mechanisms on PTC, hypothesizing that its targeted light therapy could modify PTC pathology and preserve thyroid health. We explored the application of this non‐invasive approach to expand treatments for PTC and investigated the translational medicine possibilities of light perception within the thyroid gland. The thyroid's newfound role as a light‐perceived organ provides novel insights into endocrine light responses. Our results suggest that PBMT selectively targets PTC, indicating a potential shift in treatment methods. We have shown PBMT's effectiveness in reducing PTC growth both in vitro and in vivo, affirming its potent antineoplastic benefits without the side effects of conventional treatments. PBMT's safety and efficacy offer a promising non‐invasive alternative to thyroidectomy. We introduce an optical wearable device that has completed animal testing, enhancing PBMT's practicality and potentially bridging the gap from the laboratory to the bedside.

## 2 MATERIALS AND METHODS

### 2.1 Animal

Inbred immunodeficient female BALB/cNj‐Foxn1nu/Gpt mice (BALB/c‐nu), aged 4 weeks and weighing 10–12 g, were used for tumor xenograft experiments after a one‐week acclimatization period. All animals were housed under specific pathogen‐free conditions with controlled temperature (18–22°C), humidity (50%–60%), and a 12‐h light/dark cycle (8 a.m–8 p.m). They had ad libitum access to standard rodent chow and autoclaved tap water. Mice were sourced from Nanjing GemPharmatech. For the evaluation of PBMT efficacy in the ectopic tumor model, mice were randomly assigned to the following treatment groups: Control (CTL), Red Light (RL), Green Light (GL), and Blue Light (BL), with three mice per group. Randomization was performed using a computerized random number generator to ensure unbiased allocation. Tumor cells were injected subcutaneously, and PBMT treatments were administered according to the experimental protocols. In the in situ tumor model, developed under ultrasound guidance to better replicate the human PTC environment, mice were randomly allocated to two groups: CTL and BL, with five mice per group. Randomization for the in situ model was also achieved using a computerized random number generator. Tumor volume, fluorescence intensity, and histological assessments were conducted to evaluate PBMT efficacy, while physiological parameters such as body weight, food and water consumption, and serum markers were monitored to assess treatment safety. Additionally, for the in vitro safety evaluation, primary thyroid cells were isolated from 12‐week‐old female SD rats, with three rats per group, and all procedures were carried out in accordance with ethical guidelines for animal research.

### 2.2 Ethics approval statement

All animal procedures in this investigation conform to the Guide for the Care and Use of Laboratory Animals published by the USA National Institutes of Health (publication No. 85‐23, revised 1996) and the approved regulations set by the Laboratory Animal Care Committee at China Pharmaceutical University (Permit number SYXK‐2021‐0011, 2021/01/25).

### 2.3 Cell culture

The human TPC‐1 (ATCC, USA), B‐CPAP(Cas9x, China), and Nthy‐ori 3‐1 (ATCC, USA) cell lines were purchased from the American Type Culture Collection. For the TPC‐1 and B‐CPAP cell lines, the cells were grown routinely in DMEM (Gibco, #8119007, Waltham, USA) supplemented with 10% v/v heat‐inactivated fetal bovine serum (FBS, Gibco #15140122, Waltham, USA), and 100 U/mL PenStrep at 37°C and 5% CO_2_. For the Nthy‐ori 3‐1 cell line, the cells were cultured in RPMI 1640 supplemented with 10% FBS, 1% sodium pyruvate, and 100 U/mL PenStrep at 37°C and 5% CO_2_.

### 2.4 Optical cell culture incubator setup and PBMT protocol

Design and Construction: Custom‐designed optical cell culture incubators equipped with a programmable LED lighting system (5 W, Xuzhou Ai Jia electronic technology Co.LTD) were used. LEDs covered different spectrum of wavelengths, including red (λ_R_ = 650 nm), green (λ_G_ = 520 nm), and blue (λ_B_ = 465 nm). And white light, all light kept Ee = 2 mW/cm^2^. The Optical Cell Culture Incubator ensured uniform light distribution within cell cultures. The output of each LED was calibrated for intensity and wavelength using a validated spectroradiometer (Hopoocolor Technology, China). PBMT Treatment: Cells were exposed to a predetermined irradiance of 2 mW/cm^2^ for various durations to achieve different energy exposures (Energy = 7.2 J/h). CTL groups were not exposed to light.

### 2.5 Cell viability and proliferation assays

For the EdU staining assay, TPC‐1 or B‐CPAP cells were seeded onto coverslips in six‐well plates at a density of 8 × 10^5^ cells per well and allowed to attach for at least 12 h. Cells were then treated with different wavelengths of light, including red (λ_R_ = 650 nm), green (λ_G_ = 520 nm), and blue (λ_B_ = 465 nm), at an intensity of 2 mW/cm^2^ for various durations at least 6 h (6 h = 43.2 J, 12 h = 86.4 J, 24 h = 172.8 J, 36 h = 259.2 J, 48 h = 345.6 J). After treatment, the cells were incubated with 10 μM EdU (C0075S, Beyotime) for 2 h. Cells were fixed with 4% paraformaldehyde for 15 min, permeabilized with 0.3% Triton X‐100, and subjected to Click‐iT™ EdU staining following the manufacturer's instructions. The stained cells were counterstained with Hoechst 33342 (C1025, Beyotime). Fluorescent images were captured with a Nikon microscope (ECLIPSE, Ts2R‐FL, Tokyo, Japan).

CCK‐8 Viability Assay: To assess the inhibitory effects on the proliferation capacity of thyroid papillary carcinoma cells, we conducted a wavelength‐ and dose‐dependent evaluation using the CCK‐8 method. In brief, 5 × 10^3^ cells were seeded in each well of a 96‐well plate and incubated overnight at 37°C. To synchronize the cell cycle, cells were incubated with serum‐free DMEM for 2 h. Following this, the cells were exposed to different optical environments in 100 μL of serum‐free DMEM for 24 h. Subsequently, 10 μL of CCK‐8 reagent (G021‐1‐1, Njjcbio) was added to each well, followed by further incubation at 37°C for 1 h. Finally, absorbance at 450 nm was measured using a microplate reader.

Crystal Violet Staining: TPC‐1 or B‐CPAP cells were seeded in six‐well plates at a density of 8 × 10^5^ cells per well and allowed to attach for at least 12 h. Cells were exposed to different light wavelengths and durations as described above. Following treatment, cells were washed with phosphate‐buffered saline (PBS) and fixed with 4% paraformaldehyde for 15 min. Fixed cells were stained with 0.1% crystal violet solution (CAS#548‐62‐9, Aladdin) for 20 min at room temperature. Excess crystal violet dye was washed off with distilled water, and the plates were air‐dried. Images of stained cells were captured using a Nikon microscope.

### 2.6 Flow cytometric analysis

Cell Cycle Analysis: After TPC‐1 or B‐CPAP cells were exposed to the specified PBMT, TPC‐1 or B‐CPAP cells were harvested using 0.25% trypsin solution without EDTA solution, and cell suspensions were prepared in basic DMEM medium. Cells were counted and an aliquot containing 1 × 10^6^ cells was centrifuged at 300 g for 5 min. The cell pellet was resuspended in 75% ice‐cold ethanol and fixed at −20°C for at least 12 h. For staining, cells were washed twice with ice‐cold PBS and then incubated with 500 μL of PI (propidium iodide)/RNase staining buffer (50 μg/mL PI and 100 μg/mL RNase in PBS) for 30 min at 37°C in the dark. Stained cells were analyzed using a flow cytometer (G019‐1‐1, Njjcbio). The cell cycle distribution was evaluated by measuring the DNA content in the G0/G1, S, and G2/M phases. A minimum of 10,000 events were recorded for each sample.

Cell apoptotic assay: TPC‐1 cells were treated with the specified PBMT as before. Both adherent and floating cells were collected post‐treatment. Cells were centrifuged at 300 g for 5 min, and the cell pellet was resuspended in 1 × binding buffer. The cell suspension was incubated with 5 μL of Annexin V‐FITC and 5 μL of PI for 15 min at room temperature in the dark, following the manufacturer's protocol of the Annexin V‐FITC Apoptosis Detection Kit (G003‐1‐2, Njjcbio). The stained cells were analyzed immediately using flow cytometry. The percentage of early apoptotic (Annexin V‐positive, PI‐negative) and late apoptotic (Annexin V‐positive, PI‐positive) cells was determined.

### 2.7 Establishment of 3D cell cultures

TPC‐1 cells were mixed with a Matrigel matrix (Corning) in a 1:1 ratio and seeded at a density of 3000 cells per well in 24‐well plates. The 3D cultures were maintained for 14 days, with the medium being changed every three days. During the 14‐day 3D culture, the cells underwent specific PBMT treatments. An Light Emitting Diode (LED) light source delivering 2 mW/cm^2^ intensity was used, covering red light (RL, λ_R_ = 650 nm), green light (GL, λ_G_ = 520 nm), blue light (BL, λ_B_ = 465 nm), and a CTL group. The cells were exposed to the light for 3.4 h daily, continuing this regimen for a total of 7 days (the first 7 days were the 3D cell forming period), amounting to an approximate total energy exposure of 173 J. The light exposure was administered from above the culture plates to ensure uniform light distribution. Spheroid growth and morphology were monitored daily using an inverted microscope (ECLIPSE Ts2R‐FL, Nikon, Japan). Spheroid diameters were measured using ImageJ 1.53a image analysis software. Tumor volumes were calculated using the average of the length multiplied by the square of the width. Growth curves were plotted to assess proliferation rates based on these measurements.

### 2.8 In vivo assay

Ectopic Tumor Model: We established a human TPC‐1 thyroid papillary carcinoma model using BALB/C‐NU mice (*n* = 3). This was achieved by subcutaneously injecting 5 × 10^6^ TPC‐1 cells into the neck region of the mice. After 14 days post‐injection, the mice were randomly divided into five groups, including a CTL group and different treatment groups (NFC group, RL group (650 nm), GL group (520 nm), and BL group (470 nm)). PBMT was administered at an irradiance intensity of 10 mW/cm^2^ for 15 min per day for 21 consecutive days. Fluorescence data of the tumors (TPC‐1*‐Luc*) were measured every 3 days using an In Vivo Imaging System (IVIS SpectrumCT, PerkinElmer) for the duration of 21 days. After 21 days of PBMT treatment, the mice were sacrificed, and major organs, serum, and tumor samples were collected for further analysis.

In situ Tumor Model: Similarly, we used the aforementioned method to establish an in situ tumor model by injecting 5 × 10^6^ TPC‐1 cells into the thyroid region of BALB/C‐NU mice under the guidance of a high‐resolution small animal ultrasound and photoacoustic imaging system (Vevo LAZR, VisualSonics, Fuji, Japan) (*n* = 5). After 14 days, tumor‐bearing mice were randomly assigned to a control group (CTL) and a blue light treatment group (Blue Light). Similar to the ectopic model, we recorded PTC fluorescence data, body weight, food intake, water consumption, etc., every 3 days. At the end of the treatment, CT scanning was used to record morphological changes in the tumors. Subsequently, the mice were sacrificed, and major organs, serum, and tumor samples were collected for further analysis.

### 2.9 Western blot analysis

PBMT‐treated TPC‐1 cells were lysed using Radio Immunoprecipitation Assay buffer (RIPA) buffer (89,901, Thermo Fisher Scientific) supplemented with Cocktail (W060‐1‐1, Njjcbio) and Phenylmethanesulfonyl fluoride (PMSF) (CAS#329‐98‐6, beyotime). Lysates were centrifuged at 14,000 g for 15 min at 4°C, and the supernatant was collected. Protein concentration was determined using the Bicinchoninic Acid (BCA) Protein Assay Kit (P0010S, beyotime), following the manufacturer's instructions. Equal amounts of protein were separated on Sodium Dodecyl Sulfate‐Polyacrylamide Gel Electrophoresis (SDS‐PAGE)  (10%–12%) and transferred onto Polyvinylidene Fluoride (PVDF) membranes (Millipore, Bedford). Membranes were blocked with 5% non‐fat milk in Tris Buffered Saline Tween (TBST) for 1 h at room temperature. Incubation with primary antibodies against cyclins was performed overnight at 4°C. After washing, membranes were incubated with Horseradish Peroxidase (HRP)‐conjugated secondary antibodies for 1 h at room temperature. The PVDF membranes were subjected to chemiluminescence detection, Band intensities were quantified using ImageJ software and normalized to loading controls such as β‐actin. The relevant antibody information is shown in Table S5.

### 2.10 Optical wearable devices

The main part includes four layers: a protective layer, a near‐field communication layer, a photonic transmitters layer (4.3 mm, Changzhi City Yunduoduo Co., LTD), and a polydopamine layer (CAS#51‐61‐6, Aladdin). The supporting equipment has a NFC transmitter coil directly connected to 220 V. In the synthesis of polydopamine (PDA) from dopamine hydrochloride, a 2 mg/mL dopamine solution is first prepared by dissolving dopamine hydrochloride in deionized water. This solution is then mixed in a 1:1 ratio with 50 mM Tris buffer, previously adjusted to pH 8.5. The mixture undergoes gentle stirring at room temperature, ranging overnight, facilitating the polymerization of dopamine into PDA. The resulting darkened solution indicates the formation of PDA. Post‐reaction, the PDA is separated via centrifugation at 10,000 g for 10 min, followed by repeated washing with deionized water to remove any unreacted dopamine. The final PDA product is dried under vacuum conditions. At about 60°C, the polydopamine is attached to the surface of the device.

### 2.11 Measurement of light penetration depth

To assess the penetration capabilities of the light emitted from the optical wearable device, an experiment was conducted post‐mortem on BALB/c‐nu mice. Following euthanasia, the neck region of each mouse was carefully excised while ensuring the integrity of the anatomical structure. The excised neck tissue was bisected along the trachea to create two symmetrical halves. Each half was then subjected to light exposure using the optical wearable device, positioned in close contact with the skin surface. To quantify the irradiance at various subdermal depths, a spectroradiometer (Hopoocolor Technology, China) was employed. Measurements were taken at predetermined depths beneath the skin to evaluate the irradiance levels received at different tissue layers.

### 2.12 Isolation of primary rat thyroid cells

To study the safety of PBMT, primary rat thyroid cells were isolated. DMEM/F12 medium and collagenase IV solution were preheated to 37°C. Rats were anesthetized with 4% chloral hydrate (7 μL/g body weight), and their thyroid glands were excised under sterile conditions and placed in PBS. The tissues were centrifuged at 4°C, 500 rpm for 2 min, washed three times with fresh medium, and minced into 1 mm^3^ fragments. The fragments were digested with collagenase IV solution at 37°C for 1–2 h, filtered, and centrifuged at 300 g for 5 min. Cells were resuspended in preheated RPMI 1640 medium, counted, and seeded into culture dishes with RPMI 1640 medium supplemented with 10% FBS and 1% antibiotics. Cells were incubated at 37°C, 5% CO_2_, and the medium was replaced after 24 h to ensure cell adherence. Once the primary thyroid cells adhered, they were exposed to CTL, RL, GL, and BL for 24 h to assess cell viability.

### 2.13 Co‐culture model

To evaluate PBMT safety in a more realistic pathological situation, a co‐culture system was established. Specifically, TPC‐1 thyroid papillary carcinoma cells and Nthy‐ori 3‐1 normal thyroid cells were co‐cultured in a Transwell system. In this system, 500,000 TPC‐1 cells were placed in the upper chamber, while 500,000 Nthy‐ori 3‐1 cells were seeded in the lower chamber. The TPC‐1 cells were cultured in DMEM medium containing 10% FBS, while the Nthy‐ori 3‐1 cells were maintained in RPMI 1640 medium with 10% FBS. Co‐culturing was carried out for 24 h at 37°C under 5% CO_2_ in a sterile environment. Experimental conditions: During the co‐culture period, two experimental conditions were employed: a CTL, where no intervention was applied, and a blue light treatment group (λ_B_ = 465 nm; irradiance intensity, Ee = 2 mW/cm^2^; total energy = 172.8 J). Subsequently, the supernatant from the lower chamber was collected for further analysis.

### 2.14 Statistical analysis

Statistical analyses were conducted using GraphPad Prism software (version 9.0.0). Data were expressed as mean ± SD and analyzed using one‐way ANOVA for single variables, two‐way ANOVA for interactions between two variables, and Student's *t*‐test for two‐group comparisons. The effect size was calculated to assess the significance and practical relevance of the findings. Statistical significance was set at *p < 0.05 and **p < 0.01, with “n.s.” indicating non‐significant findings. For visual simplicity, “n.s.” may be omitted in figure legends. All analyses were based on at least three independent experiments to ensure reproducibility.

## 3 RESULTS

### 3.1 PBMT wavelengths identified through cellular behavior in an optical culture system

**FIGURE 1:**
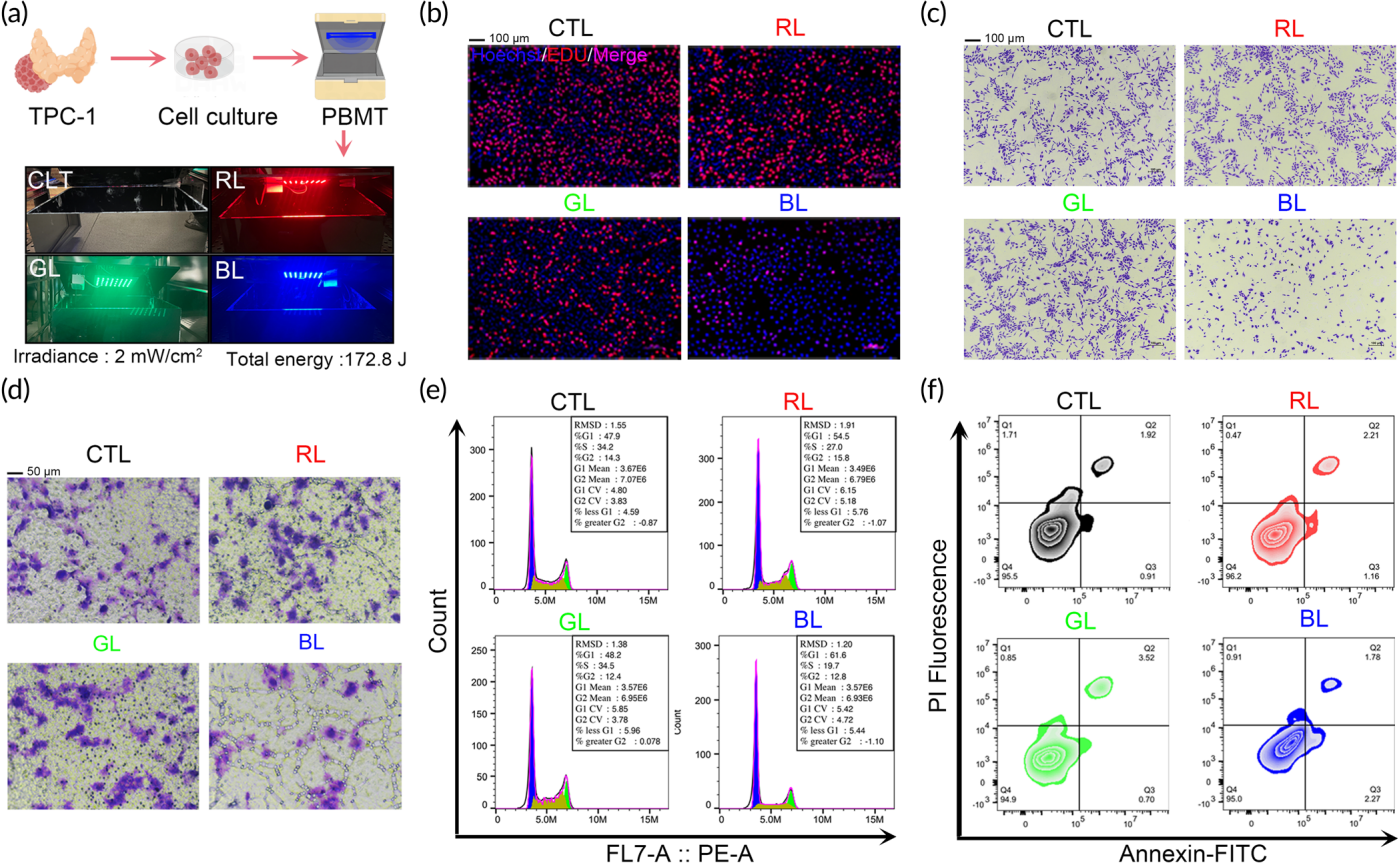
Wavelength screening and therapeutic effects of PBMT at the cellular level. (a) Schematic diagram of the optical cell culture incubator used in vitro and the experimental process flow. (b) EdU staining of TPC‐1 cells treated with CTL (Control), RL (Red light, λ_R_ = 650 nm), GL (Green light, λ_G_ = 520 nm), and BL (Blue light, λ_B_ = 465 nm). Scale bars, 100 μm. (c) Crystal violet staining of TPC‐1 cells after PBMT treatment at different wavelengths. Scale bars, 100 μm. (d) Crystal violet staining of TPC‐1 cells after transwell migration assay post‐PBMT treatment at different wavelengths. Scale bars, 50 μm. (e) Cell cycle assay of TPC‐1 cells treated at different wavelengths of PBMT. (F) Apoptosis assay of TPC‐1 cells treated at different wavelengths of PBMT.

To overcome the limitations of traditional incubators, such as the absence of customized optical devices and unstable lighting environments, an optical cell incubator was designed and constructed for this research (Figure S1A). The thyroid cancer cell line TPC‐1 was selected as the model to explore the biological effects of light on the non‐visual organ—the thyroid (Figure 1a).^33^ EdU assays revealed that blue light (BL, λ_B_ = 465 nm) significantly inhibited the proliferation of TPC‐1 cells compared to red light (RL, λ_R_ = 650 nm), green light (GL, λ_G_ = 520 nm), and the CTL (Figure 1b). Additionally, the effects of white light (which includes some blue light wavelengths) on TPC‐1 cells were investigated. The results showed that the inhibitory effect of white light on cell proliferation was intermediate between that of blue light and the control (Figure S1B,C), indicating that blue light wavelengths play a key role in the anti‐cancer effect. Crystal violet staining and CCK‐8 assays further confirmed the anti‐tumor effect of blue light PBMT, demonstrating cell morphology shrinkage and a reduction in cell number (Figures 1c, S1E). Transwell and scratch assays further validated the inhibitory effect of blue light on PTC cell migration (Figures 1d, S1F,D). To preliminarily explore the source of the anti‐cancer effect, proliferation and apoptosis of TPC‐1 cells after PBMT treatment with different wavelengths were examined. Preliminary evaluations indicated that blue light PBMT led to an increase in G0/G1 phase cell accumulation and a decrease in S phase cell accumulation, suggesting a significant blue light‐induced cell cycle arrest (Figures 1e, S1G), no effects on the apoptosis pathway was observed (Figures 1f, S1H).

To assess the safety of PBMT, primary rat thyroid cells were isolated. After treatment with PBMT at different wavelengths, no decrease in cell viability (Figure S2A) or changes in functional parameters (Figure S2B) were observed, indicating PBMT's high safety. On the other hand, in order to assess the broad applicability of PBMT to PTC and help exclude false positives, the application of PBMT was extended to another thyroid cancer cell line, B‐CPAP.^34^ A series of experiments, including EdU staining, crystal violet staining, Western blotting, and cell cycle analysis, consistently confirmed the significant inhibitory effect of this therapy on B‐CPAP cell proliferation. In the EdU fluorescence staining experiment, blue light exhibited a more pronounced inhibition of B‐CPAP cell proliferation compared to other wavelengths of light (Figure S3A). Crystal violet staining also revealed the PBMT effect in the TPC‐1 cell line, demonstrating cell morphology shrinkage and a decrease in cell number (Figure S3B). Additionally, based on preliminary investigations, the expression of cell cycle‐related proteins in the B‐CPAP cell line was assessed. The results revealed an increase in p21 protein expression and a decrease in CDK4 protein expression (Figure S3C), accompanied by cell cycle arrest (Figure S3D). These findings suggest that blue PBMT can effectively inhibit the occurrence and development of PTC while ensuring the safety of treatment.

### 3.2 Light dose‐dependent cell cycle arrest in PTC cells

**FIGURE 2:**
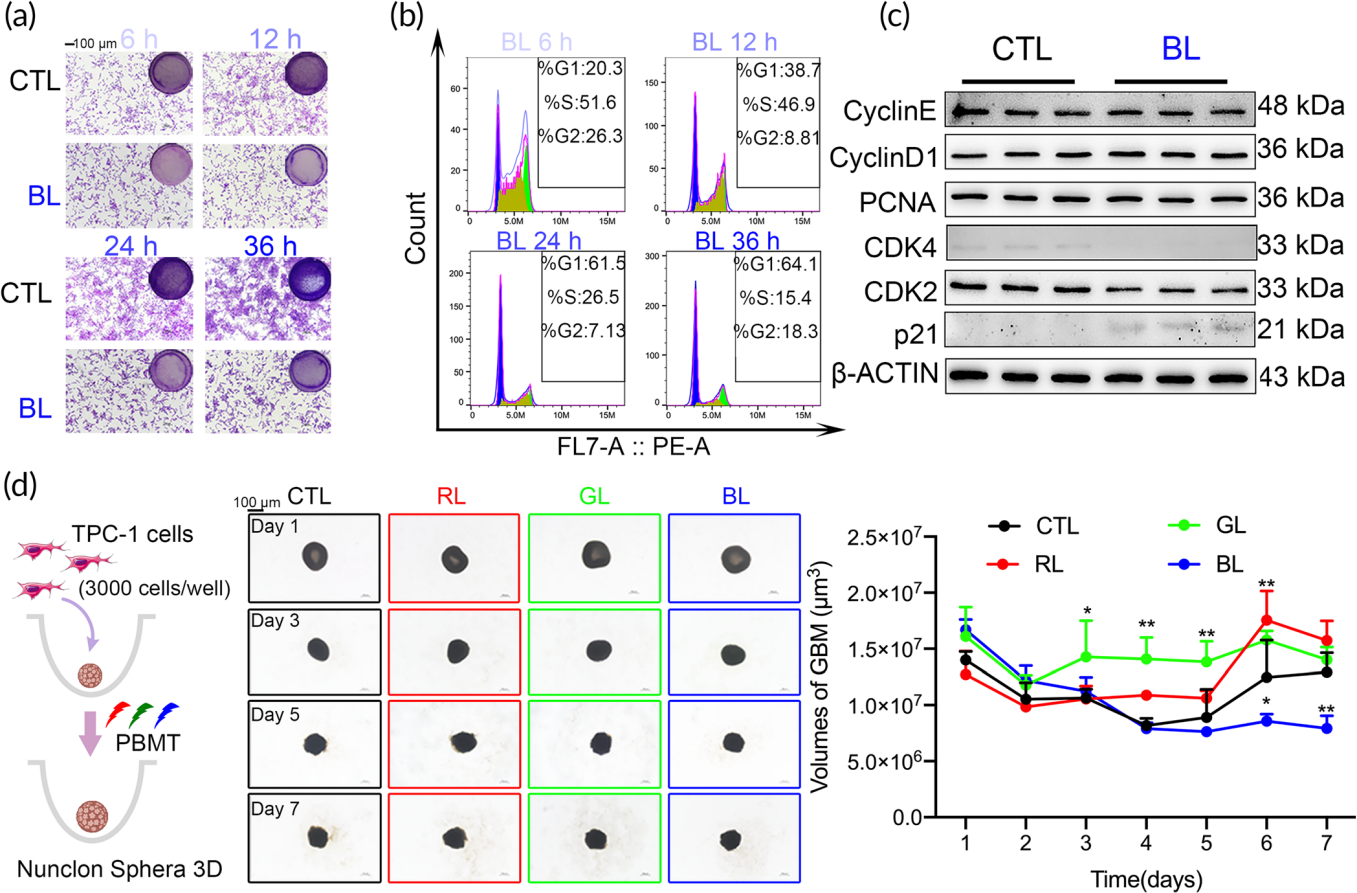
Efficacy of Dose‐Dependence on PBMT and wavelength screening at 3D cell cultures. (a) Crystal violet staining of TPC‐1 cells following treatment with different dosages of blue light (E_6 h_ = 43.2 J, E_12 h_ = 86.4 J,E_24 h_ = 172.8 J,E_36 h_ = 259.2 J). Scale bars, 100 μm. (b) Cell cycle assay of TPC‐1 cells after treatment with varying dosages of blue light. (c) Western blot analysis of cell cycle‐related proteins in TPC‐1 cells after blue light treatment(E_24 h_ = 172.8 J, *n* = 3). (d) Time‐series images and volume measurements of TPC‐1 3D cell cultures treated at different wavelengths of PBMT over 7 days (*p < 0.05, **p < 0.01, *n* = 3, vs. CTL group). Scale bars, 100 μm.

In addition to wavelength, the combination of light dose, irradiance, and exposure duration also plays a critical role in PBMT settings.^35^ After initially identifying the effective wavelengths for PBMT, the impact of light energy on the PTC cell line TPC‐1 was further explored, focusing on cell proliferation and viability. Increased light dosage resulted in reduced cell proliferation, with crystal violet staining demonstrating a dose‐dependent inhibitory effect (Figure 2a). The CCK‐8 assay confirmed that cell viability plateaued after 12 h of PBMT treatment, suggesting that the minimum effective duration of treatment is at least 12 h (Figure S4A). Flow cytometry revealed a dose‐dependent increase in cells in the G0/G1 phase and a decrease in the S phase, suggesting that under optimal irradiation of 172.8 J/cm^2^, the cell cycle arrests at 24 h (Figures 2b and S4B). These findings highlight the importance of light dosage as a critical factor in PBMT, leading to the selection of the lowest effective light dosage intensity for maximum therapeutic effect—using a 24‐h light dosage (172.8 J) in subsequent experiments. Concurrently, the expression of cell cycle‐related proteins under blue light treatment was assessed. Aberrant expressions of p21 and CDK4 proteins were observed (Figures 2c and S4C), commonly associated with cell cycle arrest in the G0/G1 phase,^36^ aligning with these findings.

### 3.3 PBMT wavelengths identified through 3D cultured PTC cells

Conventional two‐dimensional (2D) cell culture models do not adequately represent differences in light penetration, which is crucial for PBMT. To address this limitation and enhance the evaluation of PBMT's therapeutic effects, three‐dimensional (3D) cell culture bridges the gap between simple cellular models and more complex solid tumor studies. This approach is crucial for assessing the PBMT treatment in solid tumors such as PTC. To more accurately simulate the optical environment for treating PTC, our research utilized 3D cell culture techniques to construct PTC tumor models that reflect in vivo conditions. The 3D models facilitate detailed assessments of PBMT effects by featuring tumor architecture and intercellular communication. Comparative analyses demonstrated that blue light treatment significantly reduced the volume of PTC tumors in 3D cultures within 7 days. The slowed tumor volume growth highlights the therapeutic intervention's effectiveness and underscores the potential of blue light in inhibiting PTC proliferation (Figure 2d). This inhibition suggests blue light's capability to penetrate deep into tissues, preventing tumor expansion and indicating PBMT's potential for clinical translation in treating solid tumors.

### 3.4 In vivo validation of PBMT wavelengths using wearable PBMT devices

**FIGURE 3:**
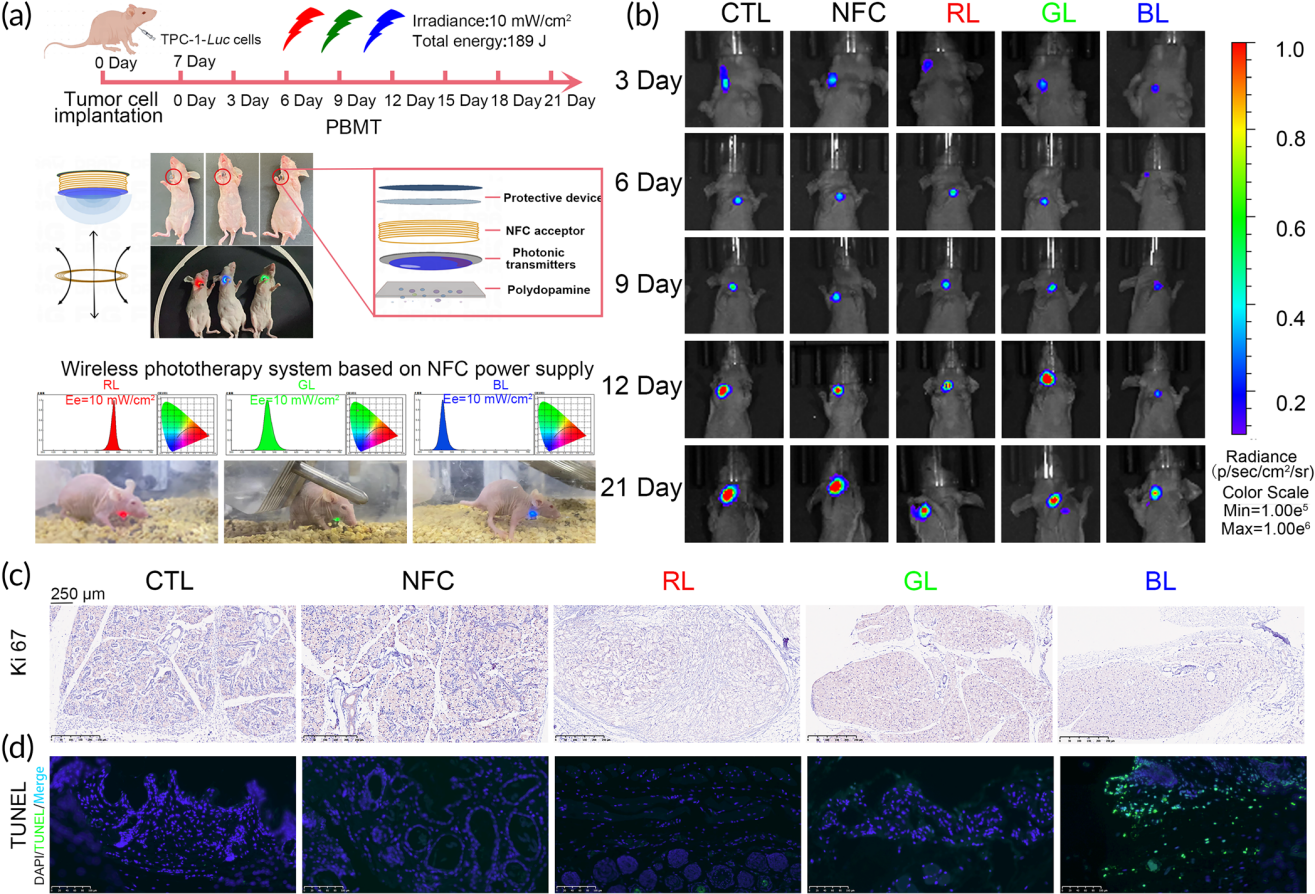
Wavelength screening and efficacy of PBMT at the animal level. (a) Diagram of the optical wearable device used in animal experiments and the PBMT treatment timeline. (b) Bioluminescence imaging of mice implanted with ectopic TPC‐1‐*Luc* tumors after treatment at different wavelengths of PBMT. Radiance Scale bars, 1.00e^5^–1.00e^6^ (p/sec/cm^2^/sr). (c) Immunohistochemical staining for Ki67 protein expression in tumor tissues treated at different wavelengths of PBMT. Scale bars, 250 μm. (d) Tunel staining of tumor tissues in ectopic tumor models after PBMT at different wavelengths. Scale bars, 100 μm.

In vivo, challenges in photobiological research were addressed by developing an optical wearable device^37^: This device overcomes issues such as visual stimulation isolation and provides wireless power through electromagnetic induction, ensuring the mobility of tumor‐bearing mice and resolving limitations associated with wired power sources. The device includes a protective structure, an electromagnetic induction receptor (NFC), photon emitters (LEDs), and a biocompatible polydopamine layer (Figure 3a). Measurements of light penetration depth confirm the delivery of light to the thyroid (Figure S5A) and provide the basis for determining the light dose through in vivo experiments. TPC‐1*‐LUC* cells were used to create an ectopic mouse model of human PTC, with tumor growth tracked in real‐time through the fluorescence intensity of the tumor cells, elucidating the therapeutic efficacy of PBMT in pathological states. Tumor fluorescence intensity was assessed thrice weekly as a proliferation indicator. The wearable PBMT device successfully demonstrated its cellular‐level therapeutic effects in the in vivo model. Over a 21‐day period, with a total energy output of 189 J, mice were exposed to different wavelengths of light (red, green, blue) from the device, as well as control and magnetic field groups, to assess PBMT effectiveness and impact (Figure 3a). Tumor growth in the blue light PBMT groups was markedly slower compared to significant growth in the control and Near‐Field Communication (NFC) groups (Figures 3b and S5B). After blue light exposure, Ki67 immunohistochemistry of tumor sections confirmed reduced proliferation (Figure 3c). However, in vivo analysis revealed apoptosis in PTC tumor tissues after PBMT (Figures 3d and S5H), indicating a stronger anti‐cancer effect by both inhibiting cell proliferation and promoting apoptosis. This suggests that PBMT might induce different cellular responses in a more complex in vivo environment compared to isolated cell cultures. Additionally, PBMT did not cause abnormalities in diet or weight, suggesting the treatment's safety (Figure S5C). To further confirm safety, histopathological evaluations with hematoxylin and eosin (H&E) staining were conducted on major organs that may be affected by the experiments. No adverse effects on the main organ systems or morphological abnormalities were observed (Figure S5D). Biochemical markers for the liver and kidneys, including Aspartate Aminotransferase (AST), Alanine Aminotransferase (ALT), creatinine (CR), and blood urea nitrogen (BUN), were also measured, revealing no hepatotoxicity or nephrotoxicity associated with PBMT (Figure S5E,F). Post‐treatment, thyroid markers T3 and T4 remained stable (Figure S5G), emphasizing the safety of PBMT and the preservation of physiological functions.

### 3.5 In situ PTC model validation reveals PBMT's therapeutic efficacy and safety

**FIGURE 4:**
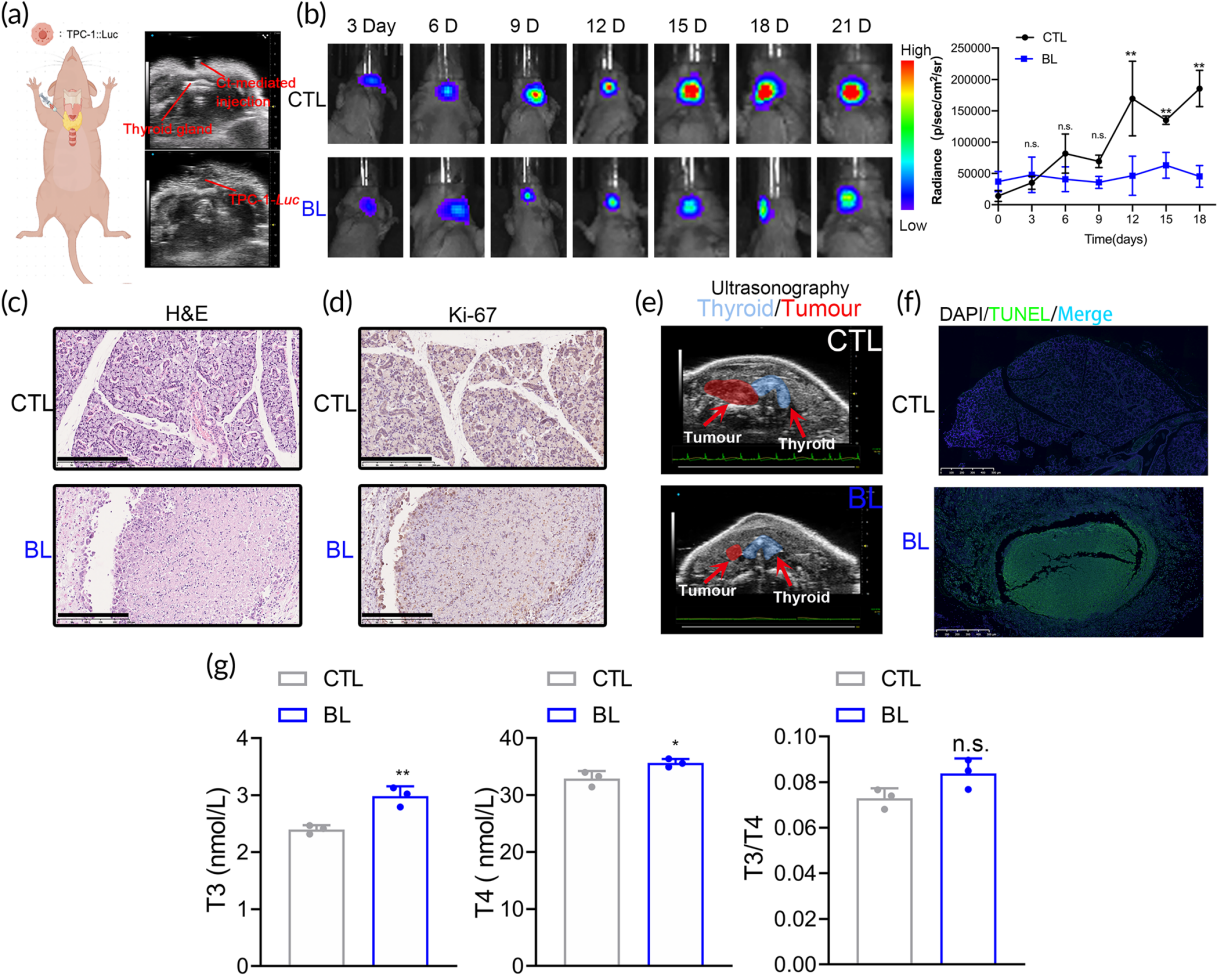
Efficacy of blue light PBMT at the animal level. (a) Images of ultrasonographic guidance to craft in situ thyroid tumor models in nude mice. (b) Bioluminescence imaging of mice with TPC‐1*‐Luc* tumors in situ after treatment with CTL and Blue light. Color scale, 150 counts‐3000 counts (n.s.: No significance, **p < 0.01, *n* = 5, vs. CTL group). (c) H&E staining of in situ tumor tissues post‐treatment with CTL and Blue light. Scale bars, 250 μm. (d) Immunohistochemical for Ki‐67 protein expression in situ tumor tissues treated with CTL and blue light. Scale bars, 250 μm. (e) Comparison of ultrasonography images of the thyroid/tumor area in CTL and Blue light groups. (f) DAPI/TUNEL double staining revealing apoptosis in TPC‐1 in situ tumor tissues after blue light treatment. (g) Changes in thyroid function markers T3 and T4 in the serum of mice with in situ tumors post‐treatment between CTL and Blue light (n.s.: No significance, *p < 0.05, **p < 0.01, *n* = 3, vs. CTL group).

An in situ nude mouse model of thyroid cancer was established under ultrasound guidance, replicating the pathophysiological environment of human PTC.^38^ This approach narrows the gap in clinical translation and allows for more accurate treatment assessment (Figure 4a). The PBMT experiments in in situ tumor‐bearing mice, mirroring the ectopic tumor protocol, demonstrated that blue light PBMT significantly inhibited the growth of situ PTC tumors (Figure 4b). After blue light PBMT treatment, H&E staining of tumor tissues from in situ PTC‐bearing mice revealed disrupted tissue structure and shrunken cell morphology in comparison to the CTL group, indicating that blue light affects the morphological characteristics of the tumor tissues (Figure 4c). Immunohistochemical staining for Ki67 protein expression showed a significant reduction in Ki67‐positive cells in the blue light treatment group compared to the CTL group within in situ tumor tissues, suggesting decreased proliferative activity of the tumor cells (Figures 4d and S6E). Ultrasonography and Computed Tomography (CT) imaging revealed a significant reduction in thyroid structure distortion induced by the tumor (Figure 4e). In the TdT‐mediated dUTP‐Nick‐End Labeling (TUNEL) assay of the situ PTC tumor‐bearing nude mouse model, similar to the ectopic tumor experiment but differing from the cellular level experiments, apoptosis was observed post‐blue light treatment (Figures 4f and S6F). ELISA tests confirmed the restoration of serum T3 and T4 levels (Figure 4g).

Consistent H&E staining of major organs and stable measurements of body weight, food, and water consumption in both in situ and ectopic tumor‐bearing mice confirmed PBMT's safety (Figure S6A,B). Furthermore, functional assessments of major organs, including kidney and liver toxicity evaluations (CR, BUN, AST, ALT), also indicated the safety of PBMT (Figure S6C,D).

## 4 DISCUSSION

This study demonstrated that blue light at 465 nm significantly curbs the growth of PTC cells more than other wavelengths. The mechanism behind this involves non‐visual photoreceptive capabilities, where cells respond to specific light wavelengths in a manner akin to how traditional vision processes light. This suggests that light can invoke biological effects similar to those seen in visual perception, but through non‐visual pathways in cells.^39^ At the molecular level, blue light interacts with opsins,^40^ such as OPN1‐SW (short‐wavelength opsin), which are known to detect short‐wavelength light. This interaction triggers a series of intracellular signaling pathways distinct from those activated by other wavelengths.^41^ For example, OPN1‐MW (medium‐wavelength opsin) and OPN1‐LW (long‐wavelength opsin) are involved in detecting medium and long wavelengths, respectively. Each of these opsins is part of the G protein‐coupled receptor family,^42^ which mediates different biological responses depending on the light wavelength.^43^

PBMT, which employs light to induce biological changes, fundamentally differs from photodynamic therapy (PDT).^44^ PDT typically involves the use of a photosensitizer, a substance that, when activated by specific wavelengths of light, transfers energy to surrounding oxygen molecules. This process initiates a photochemical reaction that produces highly reactive singlet oxygen and free radicals,^45^, ^46^ which in turn oxidize nearby biomolecules, leading to cell death or apoptosis in cancer cells.^47^ This destructive outcome is central to PDT's therapeutic effect. In contrast, PBMT does not require an external photosensitizer; rather, it leverages the body's intrinsic photoreceptive mechanisms to modulate cellular activity and activate biological pathways directly. The end effects of PBMT are primarily modulatory rather than destructive, making it a unique and non‐invasive therapeutic approach. A parallel can be drawn to the clinical success of blue light therapy in treating neonatal jaundice,^48^ which highlights PBMT's broad therapeutic potential. However, our in vivo experiments revealed an unexpected induction of apoptosis, which aligns more closely with the destructive outcomes typical of PDT. This phenomenon suggests that we may have overlooked a critical factor: the possibility that the “photosensitizers” involved in PBMT are not exclusively exogenous. The accumulation of certain endogenous substances during biological regulation could act similarly to photosensitizers, triggering apoptosis under specific conditions. Supporting this hypothesis, literature reports similar apoptotic responses in adipocytes^49^ and glioma cells^50^ following PBM treatment, which aligns with our findings. These observations warrant further investigation into the potential dual roles of light exposure in both therapeutic modulation and cellular destruction.

While this study introduces significant advancements in PTC treatment with PBMT, certain limitations and areas for further exploration must be acknowledged. A key challenge is translating results from animal models to human clinical practice. Apoptosis observed in tumor cells in vivo but not in vitro presents an anomaly, even still no apoptosis was observed in cancer cells treated with higher doses of PBMT (Figure S7). This suggests that in vivo apoptosis may be due to enhanced immune responses^51^ and interorgan communication (studies have indicated that light signals can regulate metabolic states^52^, ^53^ and the tumor microenvironment^54^), mechanisms absent in simpler in vitro systems. These findings highlight the importance of considering complex in vivo interactions when assessing PBMT's efficacy and mechanisms.

Furthermore, the long‐term effects of PBMT, particularly regarding DNA damage and the risk of secondary malignancies, require comprehensive investigation. Another factor to consider is the heterogeneity of PTC.^55^ The diverse genetic and environmental factors influencing PTC development and progression suggest that PBMT's effectiveness may vary among different patient populations. Notably, this study exclusively used female animals due to the strong gender disparity observed in PTC, with approximately 80% of patients being female. This choice aligns with the clinical prevalence of the disease and helps in more accurately reflecting the human condition. However, it is important to recognize that the results might not fully represent the effects in male subjects,^17^, ^56^ and future studies should consider this aspect to ensure broader applicability. Developing standardized protocols for PBMT is essential to ensure repeatability and effectiveness in clinical settings.^20^ Patient education and further research into user‐friendly PBMT devices are crucial for successfully integrating this therapy into routine clinical practice. Thus, while these findings are encouraging, they merely mark the beginning of a broader journey to establish PBMT as a safe and effective standard treatment.

## 5 CONCLUSION

In short, our research introduces a novel approach to treating PTC by harnessing the thyroid's unique light absorption, rooted in biomedical optics. We initially explored PBMT mechanisms in vitro, where blue light achieved anti‐tumor effects by arresting the cell cycle in the G0/G1 phase. For clinical translation, we designed a wearable device and observed effective anti‐cancer outcomes in preclinical animal studies. While clinical application challenges remain, this work marks a significant stride in PTC therapy, illustrating the transformative potential of integrating scientific innovation with biomedical optics.

## AUTHOR CONTRIBUTIONS

**Changrui Zhao:** Investigation; Conceptualization; Methodology; Validation; Visualization; Performed all experimental work. **Kun Fu:** Formal Analysis; Methodology; Visualization. **Jiameng Tian:** Investigation; Data Curation; Assisted with supplemental experiments and data analysis. **Tian Long:** Formal Analysis; Investigation; Writing—Original Draft. **Jianzhong Song:** Writing—Review & Editing; Conducted a thorough literature review and provided critical insights. **Siyu Chen:** Funding Acquisition; Writing—Review & Editing. **Chang Liu:** Funding Acquisition; Supervision; Writing—Review & Editing.

## FUNDING INFORMATION

This work was financially supported by grants from the National Key R&D Program of China (2021YFF0702000), the National Natural Science Foundation of China (92057112 and 31771298), the Natural Science Foundation of Xinjiang Uygur Autonomous Region (2023D01D05 and 2023D01D09), the Natural Science Foundation of Chongqing (CSTB2023NSCQ‐MSX0094), the Project of State Key Laboratory of Natural Medicines, China Pharmaceutical University (SKLNMZZ202214), and the Priority Academic Program Development of Jiangsu Higher Education Institutions (PAPD), the National Natural Science Foundation of China (82470930 to Chang Liu, 32471201 to Siyu Chen), and Postgraduate Research & Practice Innovation Program of Jiangsu Province (KYCX23_0876).

## CONFLICT OF INTEREST STATEMENT

The authors declare no conflict of interest.

## Supporting information


**Figure S1.** Spectral data of PBMT and validation of its therapeutic efficacy.
**Figure S2.** Safety evaluation of blue light photobiomodulation therapy.
**Figure S3.** The therapeutic effect of PBMT was verified in the same PTC cell line B‐CPAP.
**Figure S4.** Dose‐dependence of Blue Light PBMT.
**Figure S5.** Validation of PBMT safety in ectopic tumor‐bearing mice at the animal level.
**Figure S6.** Validation of PBMT safety in situ tumor‐bearing mice at the animal level.
**Figure S7.** Detection of cell apoptosis under different blue light doses in vitro.
**Table S1.** Correlation of optical parameter in vitro.
**Table S2.** Correlation of optical parameter in vivo.
**Table S3.** Analysis of effect size of cell viability assays of TPC‐1 cells following PBMT at different wavelengths.
**Table S4.** Analysis of effect size of fluorescence data in situ tumor experiment.
**Table S5.** Relevant antibody.

**Movie S1.** PBMT in vivo experiment.

**Movie S2.** Wearable photobiomodulation device.

## Data Availability

The data sets used and/or analyzed during the current study are available from the corresponding author on reasonable request.
