# Supplementary material for: Wearable photobiomodulation halts thyroid cancer growth by leveraging thyroid photosensitivity
Source: Bioeng Transl Med. 2024 Dec 20;10(2):e10734. doi: 10.1002/btm2.10734 (PMC11883128; doi:10.1002/btm2.10734)
Supplement: Supplementary file 1 — Figure S1. Spectral data of PBMT and validation of its therapeutic efficacy. Figure S2. Safety evaluation of blue light photobiomodulation therapy. Figure S3. The therapeutic effect of PBMT was verified in the same PTC cell line B‐CPAP. Figure S4. Dose‐dependence of Blue Light PBMT. Figure S5. Validation of PBMT safety in ectopic tumor‐bearing mice at the animal level. Figure S6. Validation of PBMT safety in situ tumor‐bearing mice at the animal level. Figure S7. Detection of cell apoptosis under different blue light doses in vitro. Table S1. Correlation of optical parameter in vitro. Table S2. Correlation of optical parameter in vivo. Table S3. Analysis of effect size of cell viability assays of TPC‐1 cells following PBMT at different wavelengths. Table S4. Analysis of effect size of fluorescence data in situ tumor experiment. Table S5. Relevant antibody. [file BTM2-10-e10734-s001.docx]

**Supplementary Data**
**
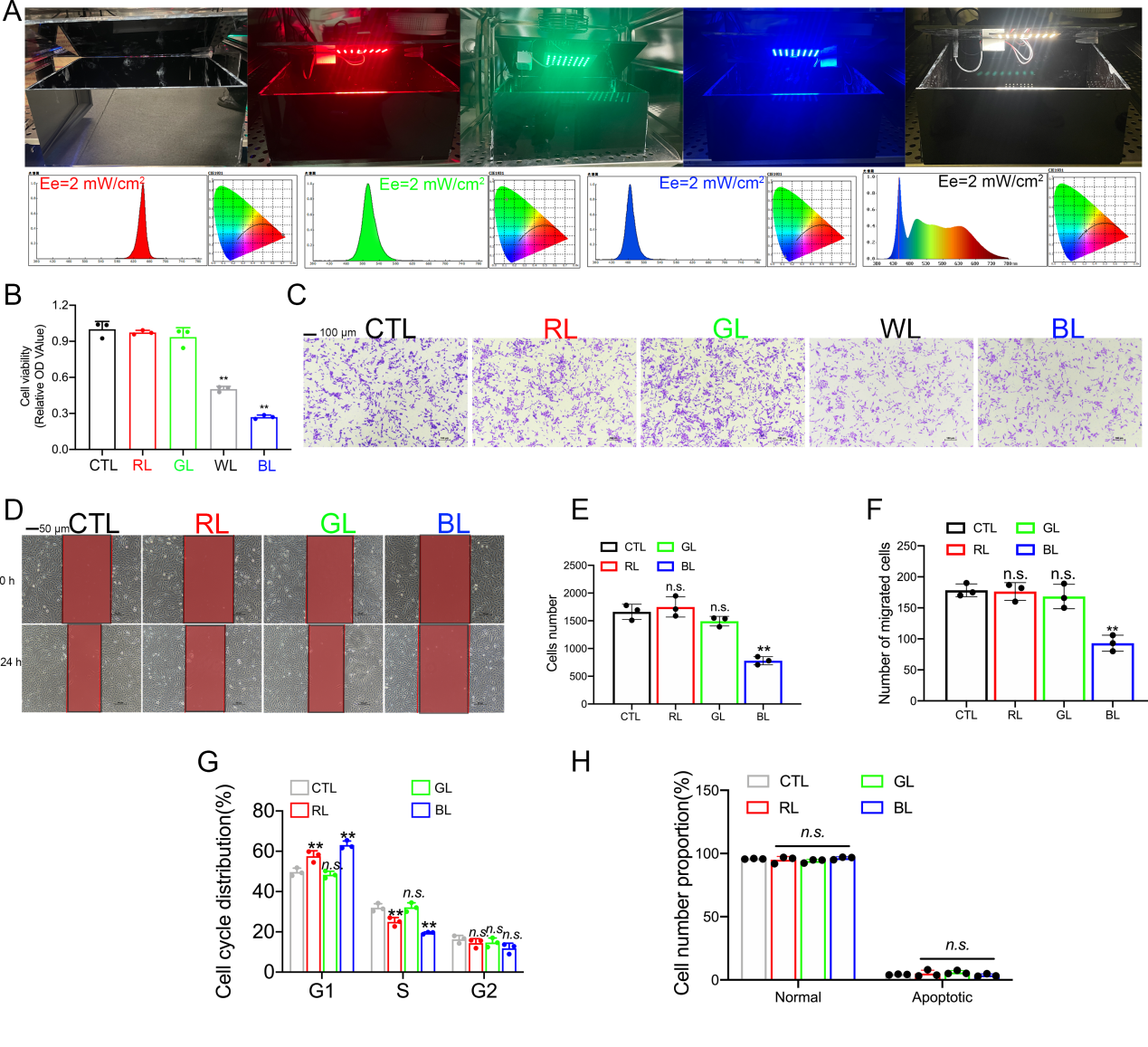
**

**Figure S1. Spectral data of PBMT and validation of its therapeutic efficacy.**

(A) Optical cell culture incubators of varying wavelengths and their spectral data.

(B) Cell viability assays of TPC-1 cells following PBMT at different wavelengths (^**^*P*<0.01, *n*=3, vs. CTL group).

(C) Crystal violet staining of TPC-1 cells after PBMT at different wavelengths. Scale bars, 100 μm.

(D) Scratch assay for invasion capability assessment of TPC-1 cells after PBMT at different wavelengths. Scale bars, 50 μm.

(E) Cell number count after PBMT at different wavelengths (n.s. : No significance, ^**^*P*<0.01, *n*=3, vs. CTL group).

(F) Number of migrated cells after PBMT at different wavelengths (n.s. : No significance, *n*=3, vs. CTL group).

(G) Cell cycle distribution analysis of TPC-1 cells after PBMT at different wavelengths (n.s. : No significance, ^**^*P*<0.01, *n*=3, vs. CTL group).

(H) Apoptosis data analysis of TPC-1 cells after PBMT at different wavelengths (n.s. : No significance, *n*=3, vs. CTL group).


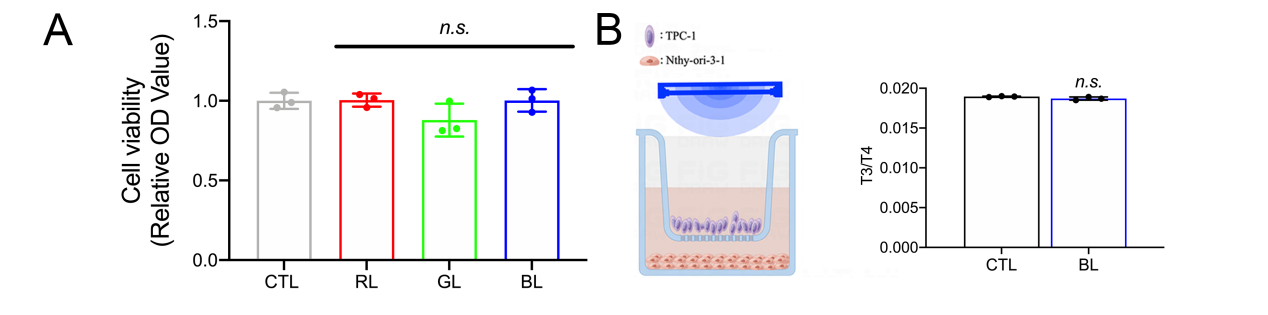


**Figure S2. Safety evaluation of blue light photobiomodulation therapy.**

(A) Cell viability assessment of primary rat thyroid cells after 24 hours of different wavelengths light treatment (n.s. : No significance, *n*=3, vs. CTL group).

(B) Evaluation of thyroid function levels (T3/T4) in co-culture systems after 24 hours of blue light treatment (n.s. : No significance, *n*=3, vs. CTL group).


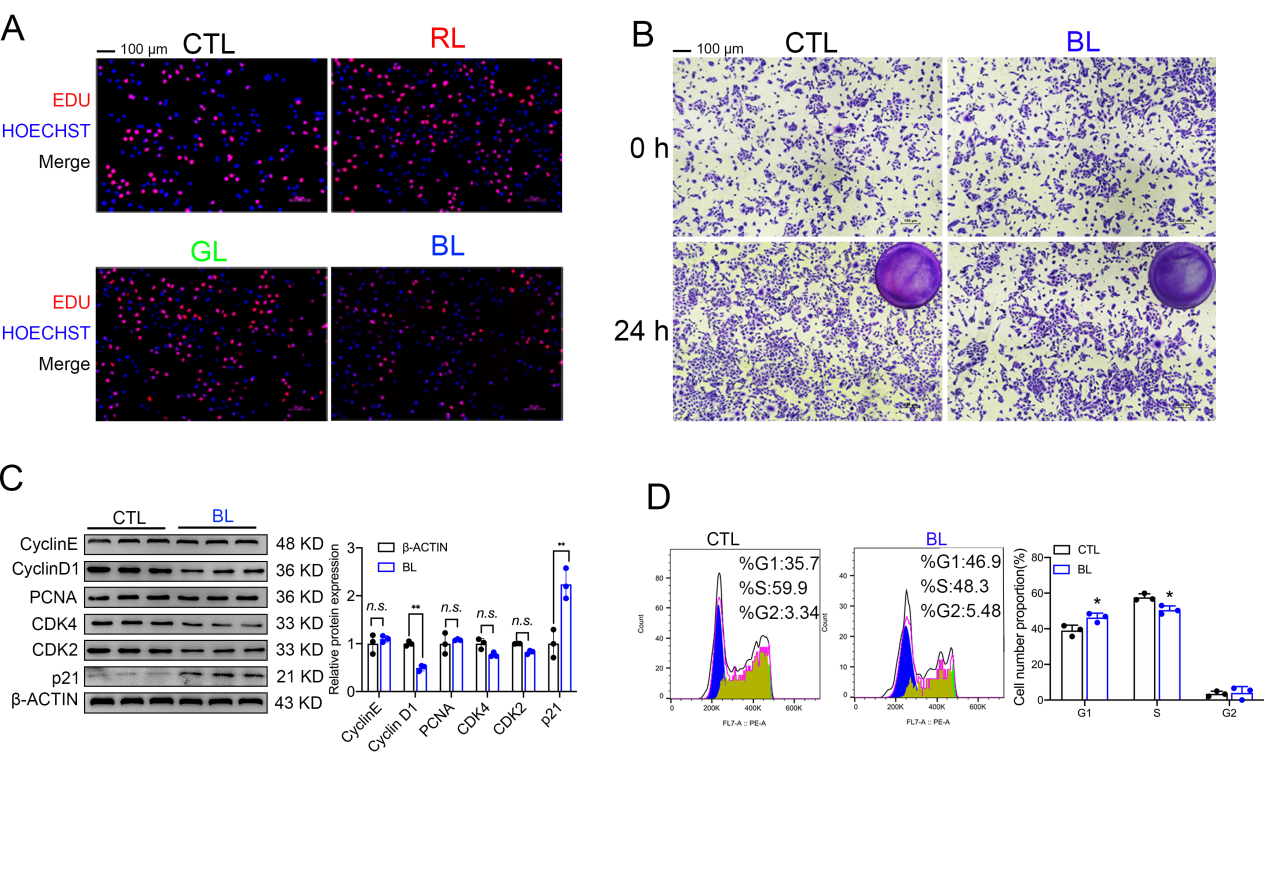


**Figure S3. The therapeutic effect of PBMT was verified in the same PTC cell line B-CPAP.**

(A) EdU staining of PTC cell line B-CPAP after 24 hours of blue light treatment.

(B) Crystal violet staining of PTC cell line B-CPAP after 24 hours of blue light treatment. Scale bars, 100 μm.

(C) Western blot analysis of cell cycle-related proteins in PTC cell line B-CPAP after 24 hours of blue light treatment (n.s. : No significance, ^**^*P*<0.01, *n*=3, vs. CTL group)..

(D) Cell cycle analysis of PTC cell line B-CPAP after 24 hours of blue light treatment (^*^*P*<0.05, *n*=3, vs. CTL group)..

**
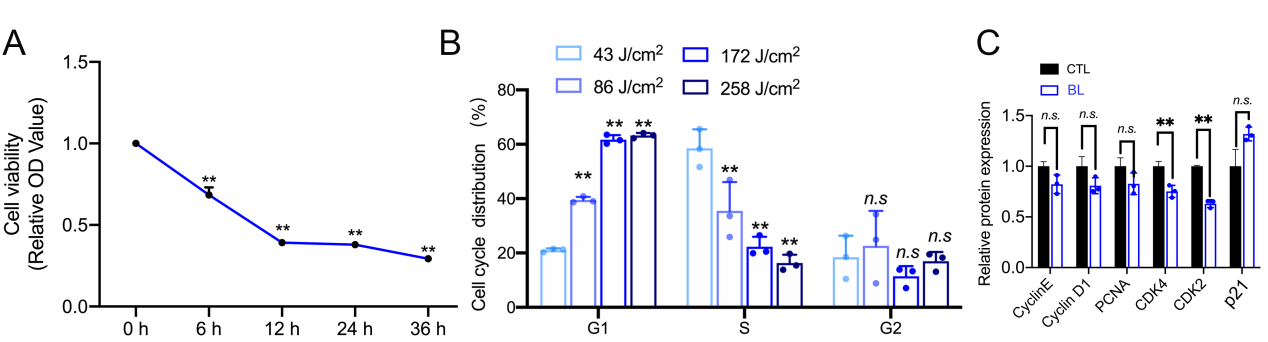
**

**Figure S4. Dose-Dependence of Blue Light PBMT.**

(A) Cell viability assays of TPC-1 cells after PBMT with different doses of blue light (^**^*P*<0.01, *n*=3, vs. 0h group).

(B) Cell cycle analysis of TPC-1 cells after PBMT with different doses of blue light (E_6 h_=43.2 J, E_12 h_=86.4 J,E_24 h_=172.8 J,E_36 h_=259.2 J, n.s. : No significance, ^**^*P*<0.01, *n*=3, vs. 43.2 J group).

(C) Western blot analysis of cell cycle-related proteins after 24 hours of blue light PBMT (n.s. : No significance, ^**^*P*<0.01, *n*=3, vs. CTL group).

**
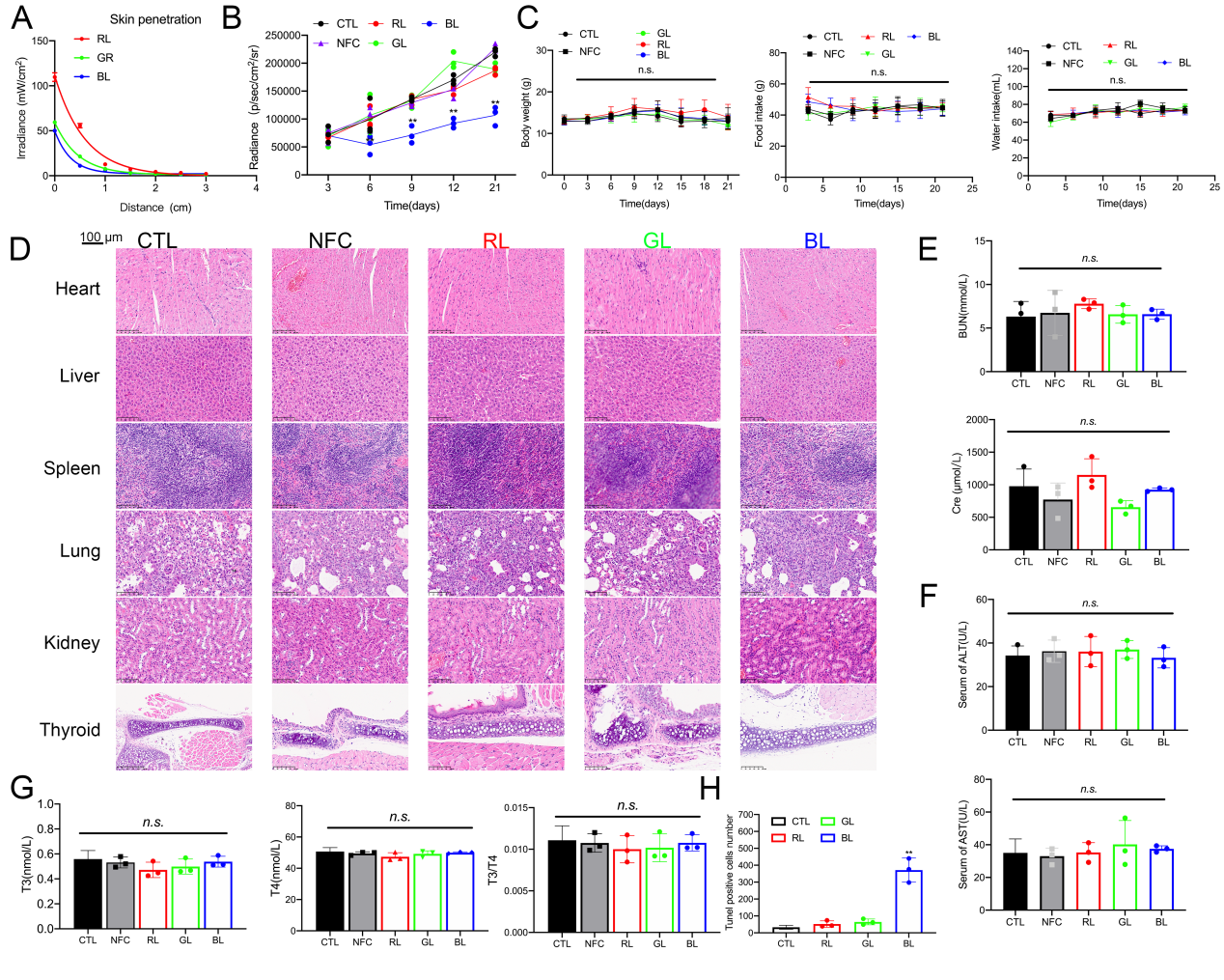
**

**Figure S5. Validation of PBMT safety in ectopic tumor-bearing mice at the animal level.**

1. Penetration depth of PBMT at different wavelengths (n=3).
2. Bioluminescence imaging of mice implanted with ectopic TPC-1-*Luc* tumors after treatment at different wavelengths of PBMT (^**^*P*<0.01, *n*=3, vs. CTL group).

(C) Body weight, food, and water intake levels after PBMT at different wavelengths in ectopic tumor models (n.s. : No significance, *n*=3, vs. CTL group).

(D) H&E staining of major organs including the heart, liver, spleen, lungs, kidneys, and thyroid after PBMT at different wavelengths in ectopic tumor models. Scale bars, 100 μm.

(E) Measurement of serum renal toxicity indicators BUN and Cre in ectopic tumor models after PBMT at different wavelengths (n.s. : No significance, *n*=3, vs. CTL group).

(F) Measurement of serum hepatic toxicity indicators ALT and AST in ectopic tumor models after PBMT at different wavelengths (n.s. : No significance, *n*=3, vs. CTL group).

(G) Measurement of thyroid function indicators T3 and T4 in ectopic tumor models after PBMT at different wavelengths (n.s. : No significance, *n*=3, vs. CTL group).

(H) Quantification of TUNEL-Positive cells after-Blue Light PBMT treatment in ectopic tumor model (^**^*P*<0.01, *n*=3, vs. CTL group).

**
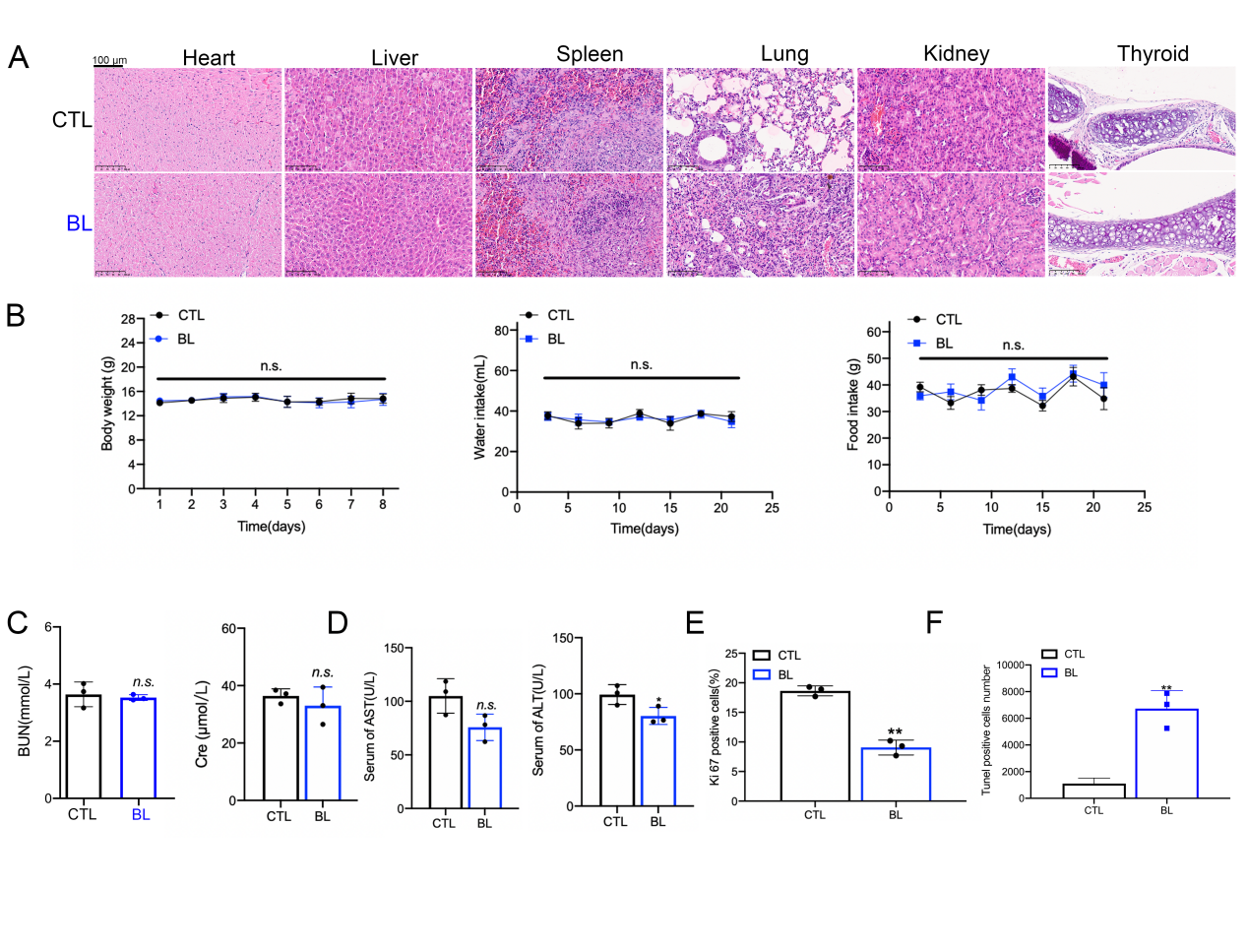
**

**Figure S6. Validation of PBMT safety *in situ* tumor-bearing mice at the animal level.**

(A) H&E staining of major organs including heart, liver, spleen, lung, kidney, and thyroid in nude mice situ tumor models after blue light PBMT. Scale bars, 100 μm.

(B) Body weight, food intake, and water consumption levels in nude mice situ tumor models after blue light PBMT (n.s. : No significance, *n*=5, vs. CTL group).

(C) Serum renal toxicity markers BUN and Cre levels in nude mice situ tumor models after blue light PBMT (n.s. : No significance, *n*=3, vs. CTL group).

(D) Serum hepatic toxicity markers ALT and AST levels in nude mice situ tumor models after blue light PBMT (n.s. : No significance, ^*^*P*<0.05, *n*=3, vs. CTL group).

(E) Proportion of Ki67-positive cells in situ tumor tissues after blue light PBMT (^**^*P*<0.01, *n*=3, vs. CTL group).

(F) Quantification of TUNEL-Positive cells after-Blue Light PBMT treatment *in situ* tumor model (^**^*P*<0.01, *n*=3, vs. CTL group).


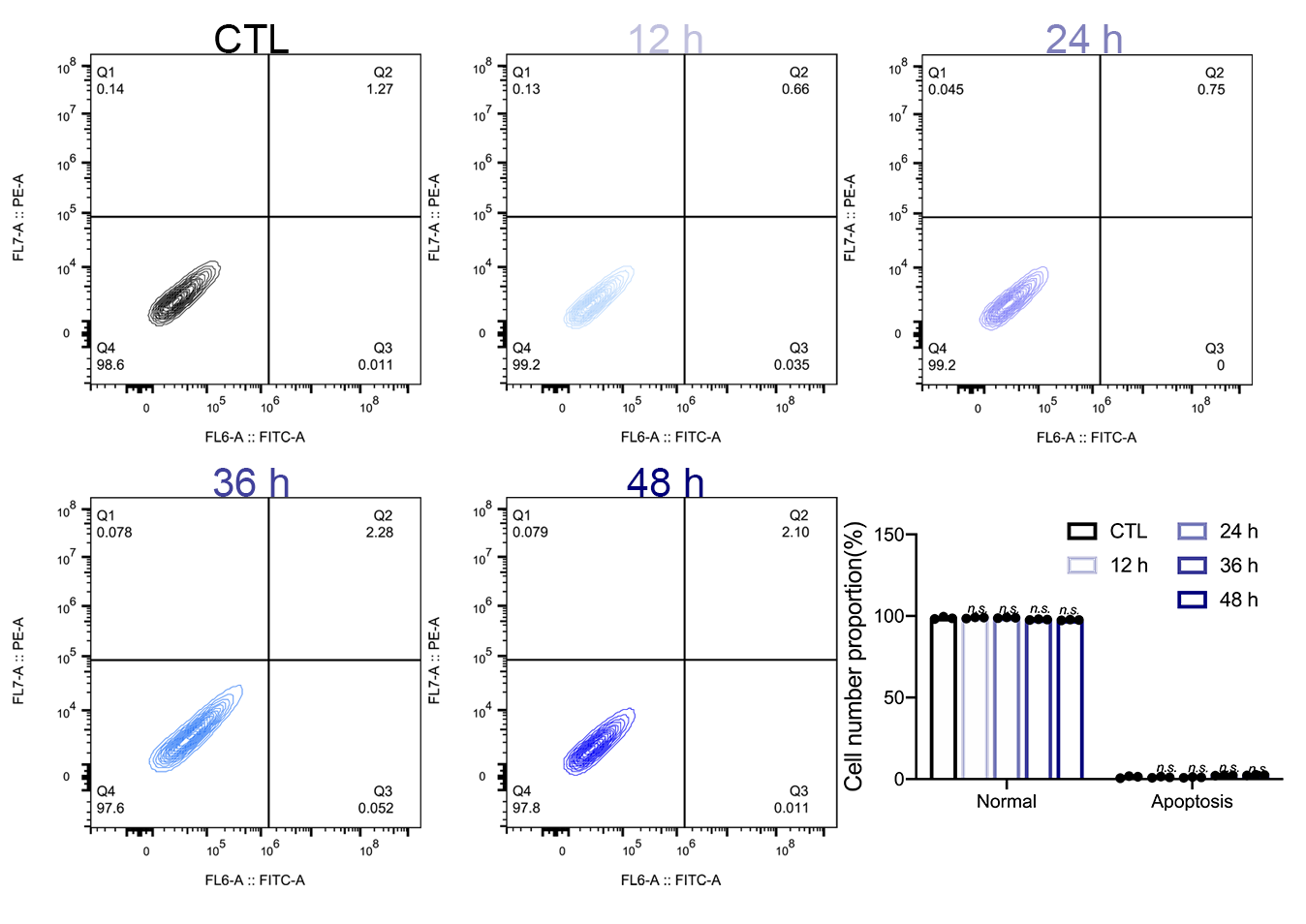


**Figure S7. Detection of cell apoptosis under different blue light doses in vitro.**

Evaluation of apoptosis after treatment with varying doses of blue light PBMT (0 h = 0 J/cm²; 12 h = 86.4 J/cm²; 24 h = 172.8 J/cm²; 36 h = 259.2 J/cm²; 48 h = 345.6 J/cm², n.s. : No significance, *n*=3, vs. 0 h Group).

| Wavelength | Exposure Time (hours) | Irradiance (mW/cm²) | Total Energy (J/cm²) | Photon Energy (eV) | Einstein Dose  (photons) |
| --- | --- | --- | --- | --- | --- |
| Blue (465 nm) | 6 | 2 | 43.2 | 2.66 | 1.01×10^20^ |
| Blue (465 nm) | 12 | 2 | 86.4 | 2.66 | 2.02×10^20^ |
| Blue (465 nm) | 24 | 2 | 172.8 | 2.66 | 4.05×10^20^ |
| Blue (465 nm) | 36 | 2 | 259.2 | 2.66 | 6.07×10^20^ |
| Blue (465 nm) | 48 | 2 | 345.6 | 2.66 | 8.10×10^20^ |
| Green(520 nm) | 24 | 2 | 172.8 | 2.38 | 4.52×10^20^ |
| Red (650 nm) | 24 | 2 | 172.8 | 1.91 | 5.65×10^20^ |

**Table S1 Correlation optical parameter *in vitro***

| Wavelength | Exposure Time  (total hours) | Irradiance (mW/cm²) | Total Energy (J/cm²) | Photon Energy (eV) | Einstein Dose  (photons) |
| --- | --- | --- | --- | --- | --- |
| Blue (465 nm) | 5.25 | 10 | 189 J | 2.67 | 4.42×10^20^ |
| Green(520 nm) | 5.25 | 10 | 189 J | 2.38 | 4.95×10^20^ |
| Red (650 nm) | 5.25 | 10 | 189 J | 1.91 | 6.18×10^20^ |

**Table S2 Correlation optical parameter *in vivo***

| Comparison  (Cell viability) | Mean CTL(%) | Std CTL  (%) | Mean Group | Std Group  (%) | Pooled SD  (%) | Cohen's d |
| --- | --- | --- | --- | --- | --- | --- |
| RL | 1 | 0.098 | 1.048 | 0.026 | 0.080 | -0.599 |
| GL | 1 | 0.098 | 1.005 | 0.014 | 0.063 | -0.076 |
| BL | 1 | 0.098 | 0.189 | 0.015 | 0.093 | 8.706 |

**Table S3 Analysis of Effect Size of cell viability assays of TPC-1 cells following PBMT at different wavelengths**

| Comparison  (fluorescence) | Mean CTL  (p/sec/cm^2^/sr) | Std CTL  (p/sec/cm^2^/sr) | Mean BL | Std BL  (p/sec/cm^2^/sr) | Pooled SD  (p/sec/cm^2^/sr) | Cohen's d |
| --- | --- | --- | --- | --- | --- | --- |
| BL | 83900.0 | 75727.0 | 1.048 | 18836.0 | 69795.2 | 0.602 |

**Table S4 Analysis of Effect Size of fluorescence data in situ tumor experiment**

| Antibodies | SOURCE | IDENTIFIER |
| --- | --- | --- |
| CyclinD1 | Proteintech | Cat##60186-1-Ig,RRID:AB_10793718 |
| CyclinE | Proteintech | Cat##11554-1-AP,RRID: AB_2071066 |
| CDK4 | Proteintech | Cat##66950-1-Ig,RRID: AB_2882273 |
| CDK2 | Proteintech | Cat##60312-1-Ig,RRID: AB_2881424 |
| p21 | Proteintech | Cat##67362-1-Ig,RRID:AB_2882614 |
| β-ACTIN | Proteintech | Cat##66009-1-Ig,RRID: AB_2687938 |

**Table S5 Relevant antibody**
